# Double-edged functions of hemopexin in hematological related diseases: from basic mechanisms to clinical application

**DOI:** 10.3389/fimmu.2023.1274333

**Published:** 2023-11-01

**Authors:** Yijin Li, Renyu Chen, Chaofan Wang, Jun Deng, Shanshan Luo

**Affiliations:** Institute of Hematology, Union Hospital, Tongji Medical College, Huazhong University of Science and Technology, Wuhan, China

**Keywords:** hemopexin, hematological diseases, systemic infections, heme, clinical

## Abstract

It is now understood that hemolysis and the subsequent release of heme into circulation play a critical role in driving the progression of various diseases. Hemopexin (HPX), a heme-binding protein with the highest affinity for heme in plasma, serves as an effective antagonist against heme toxicity resulting from severe acute or chronic hemolysis. In the present study, changes in HPX concentration were characterized at different stages of hemolytic diseases, underscoring its potential as a biomarker for assessing disease progression and prognosis. In many heme overload-driven conditions, such as sickle cell disease, transfusion-induced hemolysis, and sepsis, endogenous HPX levels are often insufficient to provide protection. Consequently, there is growing interest in developing HPX therapeutics to mitigate toxic heme exposure. Strategies include HPX supplementation when endogenous levels are depleted and enhancing HPX’s functionality through modifications, offering a potent defense against heme toxicity. It is worth noting that HPX may also exert deleterious effects under certain circumstances. This review aims to provide a comprehensive overview of HPX’s roles in the progression and prognosis of hematological diseases. It highlights HPX-based clinical therapies for different hematological disorders, discusses advancements in HPX production and modification technologies, and offers a theoretical basis for the clinical application of HPX.

## Introduction

Numerous hemolytic and thrombotic-related diseases are characterized by the excessive turnover of red blood cells (RBCs) resulting from genetic defects or acquired pathologies linked to factors such as blood transfusions, infections, and mechanical stresses. The rupture of RBC membranes leads to the release of harmful contents, including hemoglobin (Hb), heme, and arginase, among others. Hemopexin (HPX) is pivotal in the immune defense against hemolytic stress. It is a plasma glycoprotein composed of a single 60-kDa peptide chain, known for its exceptional binding affinity to heme. HPX exhibits a 1:1 binding ratio with heme at low concentrations and at least a 2:1 ratio (heme: hemopexin) at higher heme concentrations. While primarily expressed by the liver, HPX is also found in tissues such as the nervous system, skeletal muscle, retina, and kidney. It typically circulates in human plasma within a concentration range of 0.5–1.5 mg/ml (1). HPX plays a multifaceted role by sequestering free heme released from haptoglobin, participating in heme transport, and preventing peroxidation damage by induction of heme oxygenase 1 (HO-1) and metalloproteinase 1 genes (2). And it was found that HO-1 activity affects skeletal muscle aerobic capacity through heme metabolism (3).

The heme detoxification process is primarily driven by HPX through CD91/LRP1-mediated endocytosis in the liver, leading to heme degradation, reutilization, and iron metabolism. Some HPX molecules are recycled back into the plasma (4). Impaired heme clearance occurs when CD91 is saturated with its ligands or when HPX levels are depleted. Overloaded heme can bind to human serum albumin (HSA), but this complex is unstable and prone to dissociation. Free heme can penetrate plasma membranes and interact with low-density lipoproteins (LDL) (5). Heme exerts deleterious effects on endothelial function, systemic infections, and various end-organ tissues, including the lungs, kidneys, and brain. In addition to its protective role against heme toxicity, HPX mitigates methemoglobin (metHb) toxicity (6). Given its protective function against heme toxicity in hemolysis and concurrent inflammation, HPX has been investigated as a potential biomarker and therapeutic agent in heme-related pathologies and atherosclerosis. In many hemolytic diseases, HPX levels significantly decrease with disease progression, although much controversy remains regarding its classification as an acute phase reactant in humans (5, 7). Furthermore, HPX responds to certain forms of “systemic stress”. In this respect, HPX mRNA levels were notably increased in rodents subjected to sham abdominal surgery, which served as a control for partial hepatectomy (8).

Consistent with the findings of previous reviews regarding HPX’s protective role in heme-mediated pathophysiological processes, recent studies in conditions such as sickle cell disease, transfusion-induced hemolysis, sepsis, atherosclerosis, and thrombosis have validated and elaborated on the positive effects of HPX on these pathological conditions (5). However, it is important to note that this conclusion is not universally applicable, especially for systemic infections caused by certain pathogens, intracerebral hemorrhage, and Hemolytic-Uremic Syndrome (HUS), where HPX concentrations are negatively correlated with disease severity. Additionally, due to its well-established role as a heme-toxicity antagonist, significant efforts have been directed toward developing HPX-based therapies for heme-related pathologies. This review aims to provide an overview of the multifaceted roles of HPX in the pathophysiological processes of hematological-related diseases and offer an update on the latest developments in pre-clinical HPX research.

## HPX’s role in combating heme toxicity

**Figure 1:**
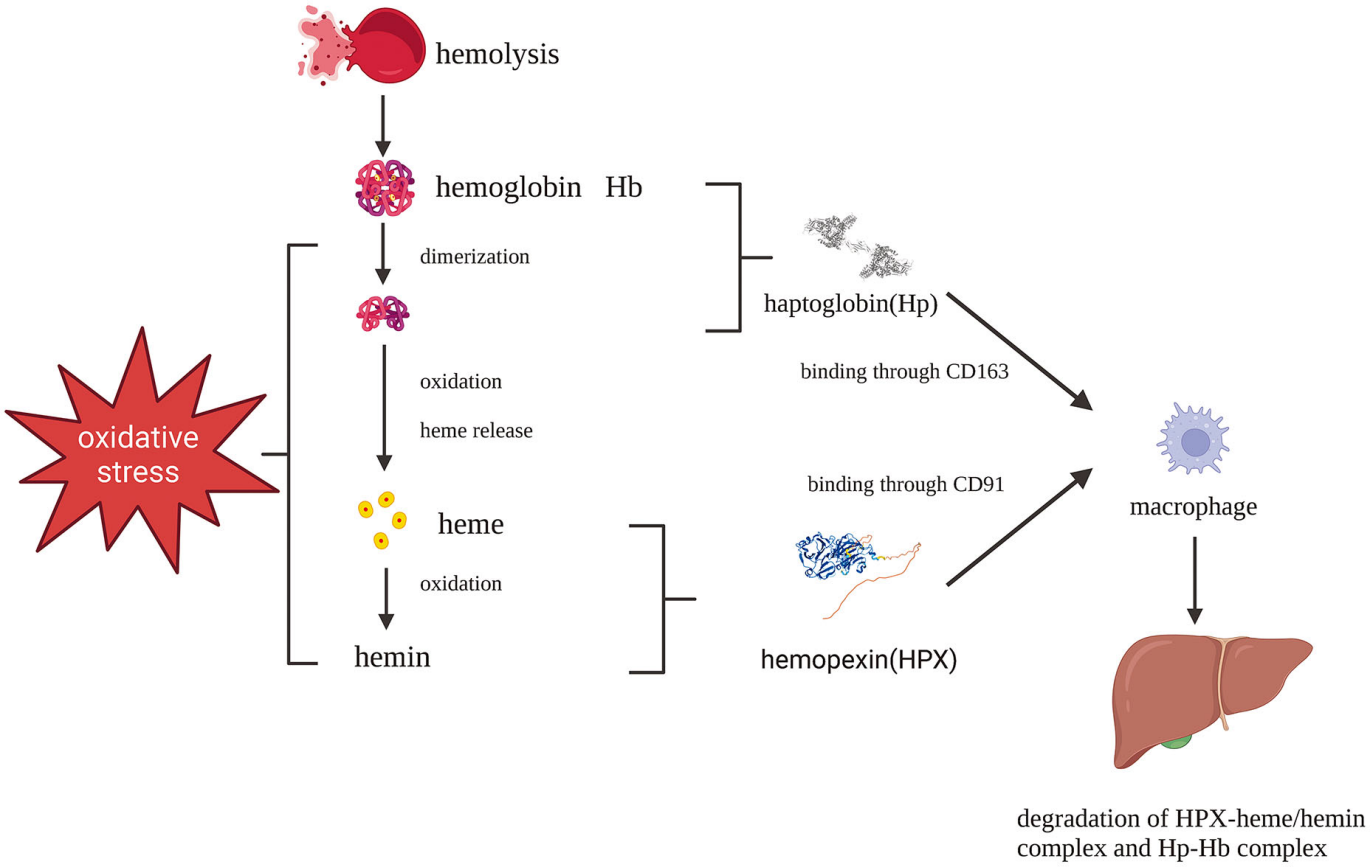
Protective pathways after hemolysis: Hb-Hp complex and heme-HPX complex fend against Hb and heme toxicity which are phagocytosed by macrophages mediated by CD163 and CD91, respectively. Created with Biorender.com.

HPX plays a pivotal role as part of the second-line defense following hemolysis. Cell-free hemoglobin (CFH) released during hemolysis is rapidly scavenged and removed from circulation primarily through the plasma protein haptoglobin (Hp) (9, 10). In various pathological conditions, such as sickle cell disease, malaria, and hemorrhage, CFH is disassociated into dimers, oxidized into methemoglobin, and quickly degraded into toxic heme (11). Structurally, heme consists of four pyrrole rings, forming an iron-protoporphyrin complex. When the iron atom is in the ferrous state, it is referred to as heme, while in the ferric state, it is known as hemin. Indeed, heme is indispensable to all living organisms but can also be a lethal molecule. Initially, heme binds to lipoproteins in the plasma before gradually transitioning to albumin and HPX (12, 13). HPX’s role in scavenging heme is a crucial step in the battle against oxidative and inflammatory disorders caused by free heme (**Figure 1**) (14). Mechanistically, the heme-HPX complex is phagocytosed by macrophages through CD91 and subsequently broken down inside the cells. Heme undergoes metabolism, forming bilirubin, carbon monoxide, and iron, facilitated by heme/Bach1/Nrf2-induced heme oxygenase-1 (HO-1). Iron is either bound to ferritin for storage or exported from macrophages to hematopoietic tissues via the iron-transporter ferroportin, where it is reused to support erythropoiesis. Free heme can activate inflammatory systems, including the complement system through the alternative pathway. Moreover, heme’s autoxidation generates reactive oxygen species (ROS) and redox-active iron, rendering it toxic to cells, tissues, and organs. As such, the clearance of heme is of utmost importance in certain hematologic disorders, especially when hemolysis depletes plasma Hp, leaving HPX as a critical second-line defense following hemolysis (2, 11, 15).

## Hemopexin’s role in hemolytic diseases

### Hemopexin’s role in sickle cell disease

Sickle cell disease is a hereditary condition resulting from a genetic mutation in HBB, which encodes the β-subunit of hemoglobin. It affects approximately 300,000 to 400,000 neonates worldwide each year (16). Current evidence suggests that hemoglobin containing mutant β-globin subunits tends to polymerize, leading to the sickling of erythrocytes, rendering them susceptible to hemolysis. The severity of SCD varies significantly and is often reflected in serum HPX levels. HPX has been identified as a mitigating factor in the pathophysiological processes contributing to the SCD phenotype, including microvascular stasis, NO scavenging, vaso-occlusion, increased levels of proinflammatory cytokines/chemokines, proinflammatory macrophages, acute kidney injury (AKI), silent cerebral infarction, immune system activation, and more. Recent studies have provided compelling evidence of HPX’s beneficial effects on SCD, as outlined in **Table 1**. For example, Vinchi F et al. discovered that HPX plays a role in preventing heme-iron accumulation in the cardiovascular system, thereby limiting the production of reactive oxygen species (ROS), increasing endothelial nitric oxide synthase (eNOS), promoting heme detoxification, and more (22). These molecular-level changes were associated with increased blood pressure and ventricular remodeling. Exogenous administration of HPX was found to nearly normalize blood pressure and improve cardiac function (22). Pulmonary hypertension (PH) is a common complication in SCD, occurring in 6-10% of the SCD population and often resulting in right ventricular dysfunction (17). Buehler et al. used an SCD murine model to simulate PH and right ventricular dysfunction, characterized by oxidation and fibrosis in SCD patients (18). They confirmed that HPX could alleviate cardiopulmonary disease in SCD mice by preventing heme-driven oxidative protein modification in the vasculature and ventricle (18). Another study revealed that chlorine inhalation triggered acute hemolysis and induced Acute Chest Syndrome in SCD mice, while post-exposure HPX administration reduced mortality and mitigated lung injury (23). In a clinical study, lower HPX levels were observed in SCD patients experiencing vaso-occlusive crises compared to patients in a steady state, further supporting the potential benefits of HPX administration in SCD (19). An additional preclinical study found that intravenously injected HPX effectively alleviated vascular stasis induced by either Hb injection or hypoxia-reoxygenation in a dose-dependent manner (20). Notably, pre-complexed HPX with heme also resolved stasis, suggesting inherent protective effects of the HPX-heme complex beyond reducing free heme levels (20). Researchers have disclosed that HPX deficiency promoted AKI in SCD, whereas HPX supplementation protected SCD mice from AKI (24, 25). Increased free heme and depletion of HPX led to the deposition of complement factor 3 (C3) and membrane attack complex in the glomeruli of patients with SCD (21). Furthermore, Poillerat V et al. found that HPX prevented heme-mediated complement activation in both plasma and the kidneys (26). In line with previous research, a recent study revealed that hemopexin could protect factor I activity *in vitro*, facilitating the degradation of soluble and surface-bound C3b, thus inhibiting complement activation and subsequent vascular and organ injury (27). Notably, Gotardo et al. developed a chronic hemolysis model in 2023, which demonstrated a decrease in Hp and HPX in accordance with chronic hemolytic anemia, thus enhancing our understanding of animal models in this field (28).

**Table 1:** Pre-clinical research on HPX and SCD.

Model	Age, Species	Treatment	Conclusions	Reference
Berkeley sickle cell mice	8-week-old; C57Bl/6 and Berk-SS mice	Subcutaneous injection of (1) saline vehicle control (n=14) (VC) (2); low dose hemopexin (LD-HPX; 100 mg/kg) (n=12); and (3) high dose hemopexin (HD-HPX, 300 mg/kg) (n=12) three times per week for 3 months.	HPX attenuates cardiopulmonary disease by preventing oxidative stress and fibrosis in the peripheral lung vasculature and oxidation in the right ventricle	(17)
SCD mice	8–16 weeks; Humanized SCD mice (SS) and humanized normal hemoglobin control mice (AA)	Cl_2_ exposure at 500 ppm for 30 min followed by either purified human hemopexin intraperitoneal injection at 10 mg/kg or vehicle with the same volume of PBS 30 min after the Cl_2_ exposure.	Post-exposure administration of hemopexin reduced mortality, plasma heme level, and RBC fragility after Cl_2_ exposure	(18)
SCD mice	12-16 weeks; Townes-SS sickle mice on a 129/B6 mixed genetic background	Injection of different doses of equimolar heme-hemopexin (5, 15, 60, and 160 mg/kg) or saline 40 min post intravenous hemoglobin injection (1 µmol/kg)	HPX dose-dependently reduces vascular stasis induced by Hb injection or hypoxia reoxygenation.	(19)
SCD mice	4-6 months; Townes expressing human Hb βS (SS) and human Hb βA (AA) mice, hemopexin-knockout mice	SS mice received oral gavage of vehicle (DMSO) or D3T 3 times per week for 3-5 months since 1 month old. Hemopexin-knockout mice were transplanted with whole bone marrow cells from SS mice (SS HPX^−/−^ and SS HPX^+/+^)	The plasma A1M to HPX ratio is associated with AKI biomarkers in SCD. HPX replacement therapy can potentially treat AKI in SCD	(20)
Phenylhydrazine-induced hemolysis	8-week-old; C56BL/6 male mice	Intravenous injection of 100 mg/kg or 500mg/kg HPX	Alleviated heme-mediated complement activation in the plasma and in the kidney.	(21)

### Hemopexin in transfusion-induced hemolysis

During transfusions, a portion of RBCs may undergo hemolysis within macrophages. Stored RBCs for transfusion undergoing “storage lesion” may compromise the quality of the blood bag, causing Hb and heme release, and adverse vascular effects including hypoperfusion and impaired endothelial function (29, 30). Transfusing RBCs stored for over 14 days has been associated with adverse outcomes in hospitalized patients (31). Despite the clear need for blood transfusions, several clinical trials and pre-clinical studies have shown that RBC administration can have detrimental effects (32, 33). Exposure to toxic substances resulting from hemolysis, including Hb, heme, and iron, is the primary cause of transfusion reactions. This results in the saturation of transferrin and reduced plasma levels of HPX and Hp, leading to the accumulation of labile plasma iron, heme, and Hb (15, 34). Brant M. Wagener et al. highlighted the crucial role of heme in the adverse effects associated with transfusions of stored RBCs (33). Restoring sequestration proteins has been suggested as a potential method to protect against tissue injury post-transfusion. A preclinical study investigated the effects of exogenous HPX on disease pathology in mice with hemorrhagic shock after transfusing stored red blood cells (SRBCs). The findings supported the protective effect of HPX in improving the survival rate and dampening the inflammatory response, although HPX did not prevent SRBC-induced hemoglobinuria and kidney injury (35). The study by Brant M. Wagener et al. demonstrated that HPX improved the survival rate of mice subjected to massive resuscitation with stored RBCs and infected with P. aeruginosa K-strain, offering evidence for HPX administration as a potential therapy to enhance the safety of SRBC transfusions. While Hp prevented hemoglobinuria, HPX showed no substantial effect (33). It is worth noting that the administration of HPX pre- or post-transfusion has been minimally investigated in clinical trials.

## Hemopexin’s role in systemic infection

### Hemopexin’s role in heme appropriation by bacteria and fungi

**Figure 2:**
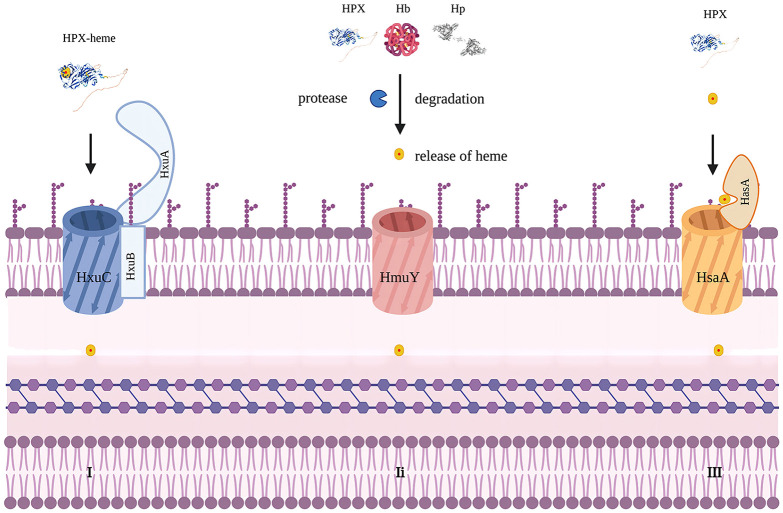
Mechanisms to acquire heme in bacteria. Modified from figure 2 in Diverse structural approaches to heme appropriation by pathogenic bacteria. This figure mainly represents HxuA/HxuB/HxuC system for heme acquirement in Haemophilus influaenzae. I. HxuA binds hemopexin and mediates heme release for import by HxuC. HxuB forms the secrete channel. II. representing HmuY system in Porphyromonas gingivalis which secretes proteases that degrade Hb, Hp and HPX and release heme for HmuY scavenging. III. representing HsaA/HsaR system in Serratia Marcescens. HsaA competes with HPX for free heme. Created with Biorender.com.

Iron is a crucial element for redox chemistry and represents a fundamental requirement for pathogens (36, 37). Transfusions of RBCs can result in increased concentrations of non-transferrin-bound iron, which can enhance pathogen proliferation and exacerbate existing infections, thus complicating the condition (33, 38). Pathogens such as *Staphylococcus aureus*, *Haemophilus influenzae*, and *Candida albicans* have developed mechanisms to release hemolytic factors that enable them to assimilate iron from hemoproteins or free heme (39, 40). They have also evolved other means to acquire iron from the host, including the expression of siderophores, hemophores, and heme uptake systems (41). For instance, *Haemophilus influenzae* can dislodge heme from hemopexin, a process facilitated by the *Hxu* operon, which encodes genes responsible for the expression of HxuA/HxuB/HxuC and is essential for extracting heme from hemopexin (42–45). *Porphyromonas gingivalis* employs HmuY as a hemophore with a distinct structure that resists proteolysis by proteases secreted for heme utilization (46). In contrast to extracting heme from host hemoproteins, *Porphyromonas gingivalis* secretes proteases to degrade hemoglobin, haptoglobin, and hemopexin, releasing heme for binding to HmuY (47). In this case, hemopexin promotes pathogen proliferation (**Figure 2**).

However, for pathogens that are unable to utilize hemopexin, it hinders iron acquisition. For example, the HasA/HasR system, which directly binds to heme, has been identified in pathogens like *Serratia* marcescens*, Pseudomonas aeruginosa*, *and Yersinia* species, potentially competing with hemopexin for free heme. A recent study found that IL-22-induced hemopexin contributes to nutritional immunity against *Citrobacter rodentium* by limiting iron acquisition by pathogens (**Table 2**) (50). In a study by Brant M. Wagener, hemopexin improved *P. aeruginosa*-induced pulmonary edema formation, post-pneumonia survival in mice undergoing massive resuscitation with stored RBCs, and reduced lung bacterial colony-forming units (CFUs) (33). HPX is also suspected of participating in the nutritional defense against *Yersinia pestis*. Higher levels of HPX induced by EV67 have been reported to prolong survival and reduce pathogen proliferation in mice challenged with a virulent strain of *Y.pestis*, making HPX a promising adjuvant therapy. The utilization of hemoglobin iron depends on a relay network of extracellular hemophores, namely, Csa2, Rbt5, and Pgt7. Specifically, Csa2 binds to hemin, while Rbt5 and Pgt7 bind to heme, facilitating heme extraction from hemoglobin and its transfer across the cell envelope (48, 49, 51). Mariel Pinsky et al. revealed that hemopexin could inhibit hemin utilization by *Candida albicans* only when the hemin concentration was lower than an equimolar concentration (52). The inhibitory effect of hemopexin was mitigated by the presence of human serum albumin (HSA), and hemin was gradually transferred to hemopexin from HSA rather than initially binding to hemopexin when released in a 1:50 HPX-HSA mixture.

**Table 2:** Pre-clinical research on HPX and infection.

Pathogens	Age, species	Treatment	Result	Reference
Citrobacter rodentium	6-8 weeks old, C57BL6 mice	IL22^−/−^, Hp^−/−^, HPX^−/−^, and Hp^−/−^HPX^−/−^ mice	IL-22-induced HPX contributes to nutritional immunity against *Citrobacter rodentium*	(46)
Pseudomonas aeruginosa	NA, C57BL/6 mice	Resuscitate trauma-hemorrhage mice with stored RBCs, followed by HPX administration and airway instillation of *Pseudomonas aeruginosa*	Reduced lung bacterial CFUs	(31)
Candida albicans	NA, *C. albicans* ccc2−/− cells	*C. albicans* ccc2-/- cells were grown for 2 days, in increasing human hemopexin concentrations in the presence of 5 μM heme, with or without 100 μM HSA	Hemopexin can inhibit hemin utilization by *Candida albicans*. This inhibitory effect of hemopexin is mitigated by the presence of HSA	(48)
Yersinia pestis	8-12weeks old, C57BL/6	Mice was injected with lethal dose of *Y.pestis* strain or together with EV67.	Post-exposure EV76 induced a rapid expression of HPX and Hp, restrained the proliferation and dissemination of *Y.pestis*, and extended survival time	(49)

NA, not applicable.

### Hemopexin’s role in sepsis

Sepsis arises from the dysregulation of the host’s immune responses to infection, leading to life-threatening organ dysfunction. Recent estimates indicate that nearly 50 million cases of sepsis were recorded worldwide, with 11.0 million sepsis-related deaths reported, accounting for 19.7% of all global deaths in 2017 (53). Although age-standardized incidents and mortality rates have decreased significantly from 1990 to 2017, sepsis remains a significant global health burden (53).

It is now understood that sepsis exposes red blood cells to various physiological stressors, increasing the risk of hemolysis and the release of cell-free hemoglobin into circulation. One of the most recognized causes of hemolysis during sepsis is disseminated intravascular coagulation (DIC), a common complication of sepsis (54). During sepsis, red blood cells also undergo deformation, driven by cytokines and pathogen-specific factors such as hemolysins produced by *Staphylococcus aureus*, *Enterococcus spp*, and *Escherichia coli* (55–57). Consistent with the process of sepsis-induced hemolysis, the plasma level of cell-free hemoglobin significantly increases in sepsis patients, positively correlated with mortality (58, 59). Accordingly, septic patients with lower hemopexin levels tend to experience death earlier than those with higher hemopexin levels. It has been found that the concentrations of hemopexin in the plasma of non-survivors are significantly lower than those in survivors with sepsis (60, 61). In pre-clinical studies (62). Larsen et al. investigated the role of heme and HPX in a polymicrobial sepsis murine model. They administered exogenous heme to low-grade polymicrobial infection mice, which markedly increased markers of organ dysfunction and sepsis severity. In cases of fatal severe sepsis after high-grade infection, reduced serum concentrations of HPX have been reported. However, when HPX was administered to mice after high-grade infection, it prevented tissue damage and lethality (62).

The protective role of HPX is attributed to its ability to neutralize the oxidative and cytotoxic effects caused by heme. To carry out this beneficial process, the expression of OH-1 is required to catabolize HPX-bound heme (62). Moreover, HPX can suppress the systemic growth of *E.coli* in *C. rodentium*, shedding light on its therapeutic potential in sepsis (50). Therefore, HPX can be regarded as a potential therapeutic strategy for preventing fatal consequences in individuals with severe sepsis (63). However, there has been a scarcity of pertinent pre-clinical and clinical trials conducted to elucidate the impact of HPX replenishment in sepsis.

### Hemopexin’s role in malaria, hemolytic-uremic syndrome, and dengue hemorrhagic fever

There were 247 million malaria cases in 2021, resulting in the loss of 61,900 lives in 2021, according to the World Health Organization (WHO). Malaria is characterized by hemolysis, which results in the release of hemoglobin and heme. The ratio of heme to HPX is inversely associated with disease severity and the 6-month mortality rate (64). Over the past 30 years, the incidence of severe dengue hemorrhagic fever cases has increased, with many patients succumbing due to a lack of timely clinical intervention. In DHF, fibronectin, HPX, and transferrin levels significantly rise in all three phases compared to Dengue Fever (DF) (65). Besides, a 2014 study revealed increased levels of HPX and vitronectin in both DF and DHF compared to healthy controls (66), suggesting HPX as a potential biomarker to distinguish between uncomplicated dengue fever and dengue hemorrhagic fever (5).

However, HPX does not always act as a protective factor in infection-related hemolysis. For instance, in a mouse model of Shiga toxin-induced hemolytic-uremic syndrome (HUS), HPX deficiency was protective in resolving HUS pathology (67). HPX^-/-^ mice exhibited higher survival rates when challenged with Shiga toxin, with reduced renal inflammation characterized by decreased macrophage and neutrophil recruitment and C3c deposition [66]. This finding aligns with a previous study by Spiller et al., which concluded that HPX deficiency was protective in sepsis (68).

## Hemopexin’s role in hemorrhagic diseases

### Hemopexin’s role in intracerebral hemorrhage

Intracerebral hemorrhage is the most common type of hemorrhagic stroke and has the highest mortality rate of all stroke subtypes (69, 70). The rapid accumulation of blood within the brain parenchyma leads to the disruption of the normal anatomy and the increase of local pressure (71–73). Heme has been identified as a proinflammatory substance in the brain, and HPX can target it effectively (73).

HPX is present in the plasma and expressed by neurons and glia in the central nervous system. Intrathecally produced HPX represents another source of HPX in the cerebrospinal fluid (CSF) (74). However, HPX levels in the CSF are approximately tenfold lower than those in circulation, suggesting a relatively low capacity for heme binding in the brain, which can become easily overwhelmed (75). An *ex vivo* study supported the neuroprotective effects of HPX, as it protects neurons and glial cells from blood-related injuries through antioxidation and downregulation of HO-1 and caspase-3 (76). Preclinical research indicated that higher levels of HPX in the brain improve outcomes after ICH in a mouse model. Elevated local HPX levels were associated with smaller lesion volumes, reduced perihematomal tissue injury, and trends toward decreased hematoma volumes (77). Additionally, mice with higher HPX levels experienced no significant changes in brain iron levels and HO-1 but exhibited increased microgliosis and decreased astrogliosis and lipid peroxidation. This suggests the potential synergy of central and peripheral heme clearance (77).

**Figure 3:**
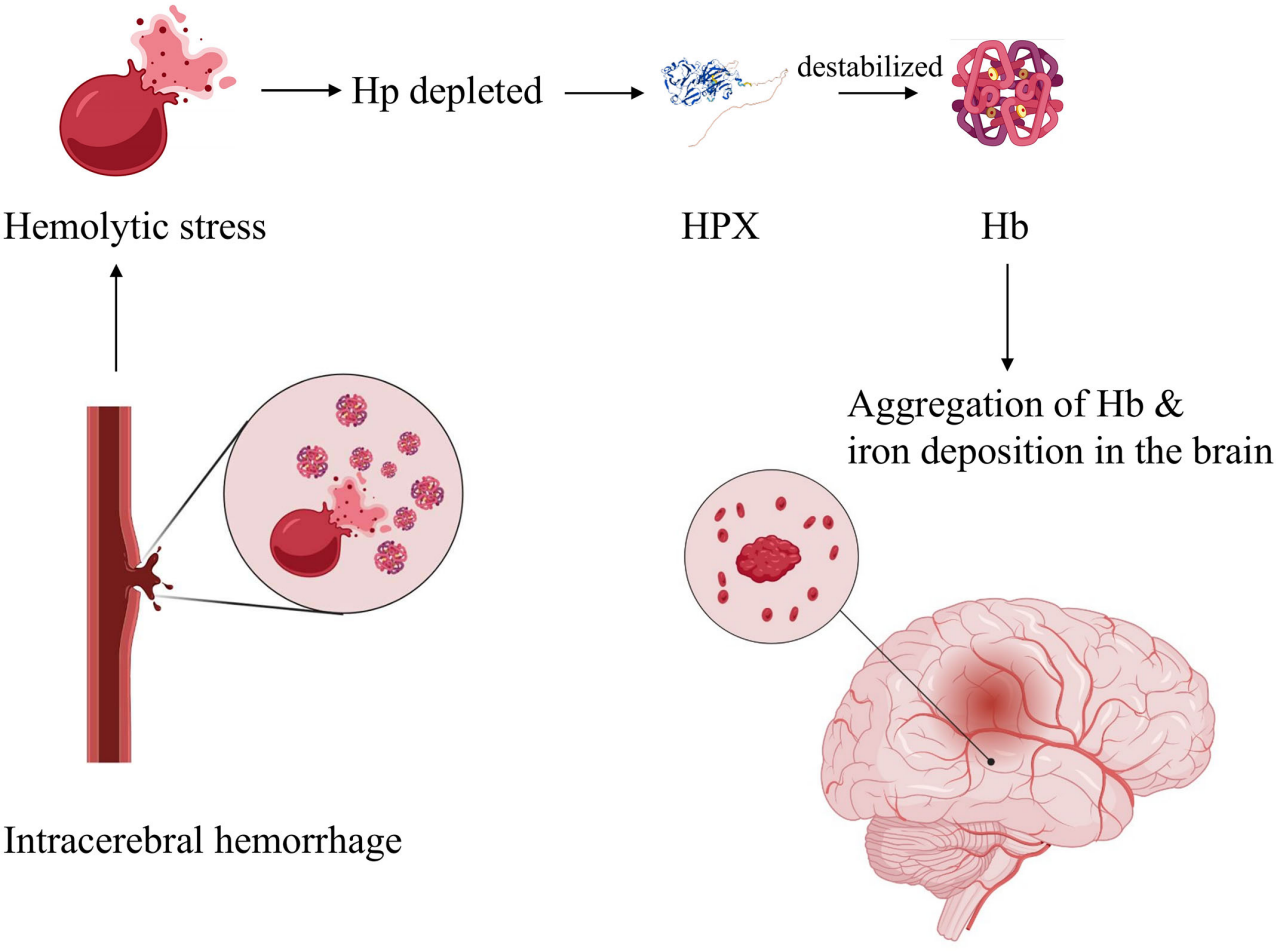
Hypothetic mechanism of globin-mediated neurotoxicity in the absence of Hp upon HPX treatment. Briefly, in the absence of Hp, application of HPX destabilizes Hb, resulting in globin precipitation and iron deposition, therefore exacerbating oxidative cell injury. Created with Biorender.com.

Co-administration of hemopexin effectively mitigated heme-derived toxicity at both molecular and cellular levels (73). However, an *in vitro* study revealed increased globin-mediated neurotoxicity when Hp was absent after hemopexin treatment. In contrast, when used in combination with Hp, the neurotoxicity was notably reduced (78). This observation supports the notion that Hp-CD163 plays an important role in activating detoxification pathways. Another hypothesis suggests that hemopexin may destabilize Hb in the absence of haptoglobin, leading to globin precipitation and iron deposition, ultimately exacerbating iron-dependent oxidative cell injury (**Figure 3**) (78). Consequently, it has been proposed that a combined therapy involving hemopexin and haptoglobin may be more favorable following intracerebral hemorrhage than a hemopexin supplement alone[77]. When administered systemically through intraperitoneal injection, hemopexin exhibited a reduction in blood-brain barrier (BBB) disruption on day 3 in ICH mouse models. However, it had no significant effect on striatal heme content on days 3 or 7, and it did not alleviate neurological deficits, inflammatory cell infiltration, or the viability of perihematomal cells on day 8 (79). This suggests that the administration of hemopexin in this manner is inadequate for allowing hemopexin to penetrate the BBB, which likely accounts for these results.

### Hemopexin’s role in subarachnoid hemorrhage

Subarachnoid hemorrhage is a type of hemorrhagic stroke primarily resulting from the rupture of saccular aneurysms, accounting for 3% of all stroke cases (80). The presence of hemoglobin and its breakdown products in the brain, combined with the toxic cascade initiated during early brain injury, has been extensively studied and is strongly linked to the development of delayed brain injury (81). In a study involving 30 SAH patients, free heme was still detectable in the cerebrospinal fluid after SAH, suggesting saturation of the HPX-CD91 system following SAH (74). Interestingly, elevated HPX levels in the CSF after SAH were associated with a higher likelihood of delayed cerebral ischemia and poorer neurological outcomes, which differed from the effects of HPX in other diseases (74). The underlying mechanism remains to be elucidated, but a possible explanation is that when the level of hemopexin in the CSF is excessively high, it may become deleterious as it impedes the efflux of heme due to its high heme affinity, resulting in intracellular heme/iron overload, which can be toxic to neurons and glia (74). It is worth noting that while bleed size and severity may be controlled, the subtypes of Hp were not in a recent review, emphasizing the need for more studies (82). In addition, CD91 is responsible for ApoE uptake, and reduced ApoE levels were associated with more severe neurological injury and worse outcomes after SAH (83–86). Thus, Hp-HPX replenishment could scavenge Hb and heme while limiting undesired ApoE uptake (82).

## Hemopexin’s role in atherosclerosis and thrombosis

Atherothrombotic diseases have long been a leading cause of death worldwide, accounting for over a quarter of all global deaths (87). Although the components involved in thrombosis and atherosclerosis differ, it is increasingly recognized that they are closely intertwined in biochemical and cellular mechanisms, particularly concerning platelet function (88). Notably, small platelets, as opposed to their larger counterparts, are rich in proteins associated with iron homeostasis, including HPX, Hp, α-1 anti-trypsin, transferrin, and vitronectin (89, 90). It is highly conceivable that small platelets take up these plasma proteins via the open canalicular system (OCS) and store them in α-granules (91). Since platelets release their contents upon activation (92), small platelets may play a role in iron homeostasis during thrombosis. *In vitro* studies have indicated that incubation of platelets with heme leads to ferroptosis and platelet activation. These effects are driven by ROS-driven proteasomal activity and lipid peroxidation and can be mitigated by melatonin. However, the role of HPX in this process requires further investigation (93, 94). It is hypothesized that the ferroptosis of smaller platelets may lead to the release of HPX, helping to mitigate ion toxicity.

Heme toxicity contributes to the progression of atherosclerosis in multiple ways. With its hydrophobic nature distributed within lipid compartments like lipoproteins, heme initiates peroxidative reactions, promoting atherosclerosis and elevating iron levels in advanced atherosclerotic lesions (95, 96). Besides, vascular endothelial injury in atherosclerosis leads to platelet adhesion and subsequent pro-thrombotic and pro-inflammatory effects. Studies have shown that HPX-related therapy alleviates endothelial activation and oxidation in SCD mice (22). After intraplaque hemorrhage, local cells were exposed to heme-induced oxidative damage, such as endoplasmic reticulum (ER) stress, which worsened atherosclerosis. An *in vitro* study demonstrated that HPX could significantly mitigate heme-induced ER stress in human aortic smooth muscle cells, indicating the protective role of HPX in atherosclerosis (97).

The protective role of hemopexin in clotting diseases has been validated by pre-clinical studies. An *in vivo* study by Wang et al. found that high doses of heme led to a dose-dependent increase in the plasma level of thrombin-antithrombin complexes, indicating an ongoing tissue factor-dependent coagulation process in SCD mice. Recombinant hemopexin treatment partially inhibited this coagulation activation (98). An *ex vivo* study revealed that heme-induced contractile dysfunction of human cardiomyocytes could be alleviated by hemopexin (99). Furthermore, in HPX-null mice with venous thrombosis induced by ligation of the inferior vena cava (IVCL), the clot size and weight were substantially greater than those in wild-type IVCL mice, underscoring the protective role of HPX in venous thrombosis (100). Both RT-PCR and Western blot analysis showed higher HPX expression in the IVCL group compared to the sham group (100). However, clinical studies on HPX therapy for these diseases are still lacking.

The timely detection and assessment of plaque presence and stability are critical, as the rupture of atherosclerotic plaques can have dire consequences. Quantitative proteomics analysis revealed that the serum concentration of hemopexin was elevated in individuals with coronary heart disease (CHD) and coronary atherosclerosis compared to healthy controls (101). Mass spectrometric analysis further indicated that hemopexin concentration was lower in serum from patients with unstable atherosclerotic plaques compared to those with stable atherosclerotic plaques, suggesting a connection between plaque stability and hemopexin concentration (102). In addition, serum proteomics analysis showed increased HPX levels on day 3 after ST-elevation myocardial infarction (103). Furthermore, a higher level of hemopexin (isotypes 1 and 2) was associated with future cardiovascular mortality in the healthy population (104). Further research into the role of hemopexin in the diagnosis and prognosis of atherothrombotic diseases is warranted.

## Clinical application of hemopexin

### Potential biomarkers to assess heme load and disease prognosis

In various hemolytic diseases, heme overload often significantly decreases HPX plasma levels, diminishing HPX’s ability to scavenge heme effectively. Identifying an increase in plasma heme levels can aid in diagnosing and developing targeted plasma-based therapeutics (5). The level of HPX is considered a biomarker to assess heme load in many hemolytic pathologies. It was first proposed in 1975 that plasma HPX levels could indicate the severity of hemolysis, and this parameter has since been established to assess the level of hemolysis. For instance, in SCD, HPX levels in plasma increased as hemolysis levels declined in response to hydroxycarbamide treatment (105). Patients with septic shock who experienced lethal outcomes and severe organ injury were found to have reduced HPX concentrations (62, 64). In the case of malaria, the heme to HPX ratio negatively correlated with disease severity, as estimated by severe anemia, respiratory distress, stage 3 acute kidney injury, and the 6-month mortality rate (56). However, the levels of HPX increased significantly in all three phases of dengue hemorrhagic fever, which contradicts serum HPX changes in several other diseases. A higher level of HPX in the CSF was associated with better outcomes in ICH but worse prognosis in SAH (74, 77). The next step may involve measuring HPX levels at different stages of hemolytic diseases to further establish its role in prognosis and treatment in clinical scenarios.

### Hemopexin replenishment therapy

**Figure 4:**
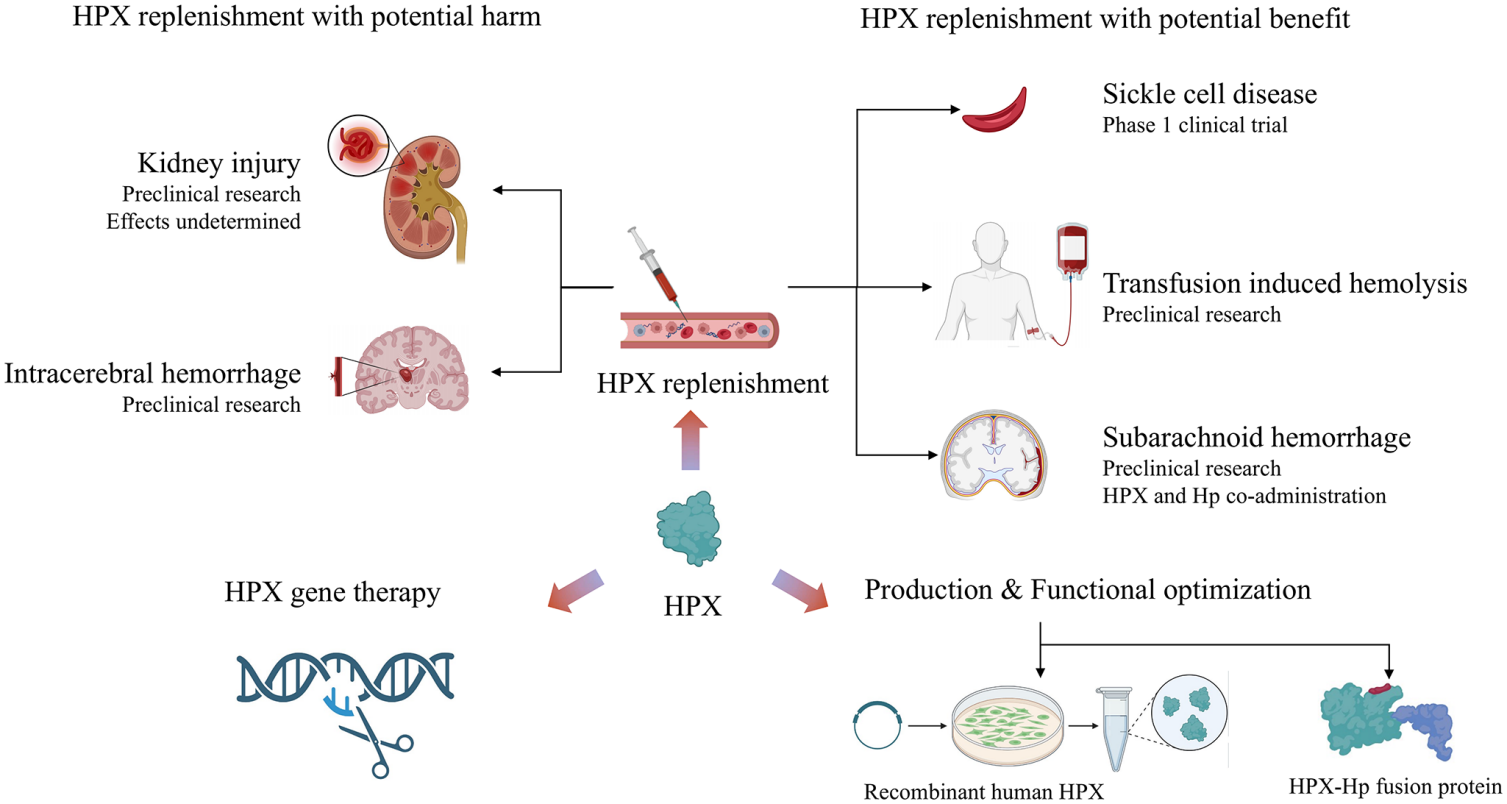
Summary of current research on the therapeutic potential of HPX: HPX replenishment therapy shows double-edged function in homological disorders. Novel directions include production and functional optimization of HPX, as well as HPX gene therapy. Created with Biorender.com.

Considering an average plasma concentration of 770 μg/ml of HPX in adults, each milliliter of plasma can bind 6.3 μg of heme, and higher heme levels can deplete HPX unless recycling or rapid compensatory synthesis occurs (1). As discussed earlier, HPX has been evaluated as a therapeutic agent in various heme overload diseases, including SCD, blood transfusions, sepsis, malaria, hemolytic-uremic syndrome, ICH, and SAH. The preponderance of data supporting HPX as a therapeutic target suggests that HPX replenishment remains an adjuvant therapy for hemolytic disorders with high heme stress (**Figure 4**). Over the years, preclinical models have been employed to evaluate potentially applicable scenarios. Notably, a phase 1 clinical trial is investigating HPX dosage in patients with sickle cell anemia, focusing on safety, tolerability, and pharmacokinetics (NCT04285827). However, HPX administration has only been approved for SCD by the European Commission and FDA in 2020 (20).

Beyond its application in SCD, preclinical trials have provided proof of concept for using HPX in other clinical settings. For example, in transfusion-induced hemolysis, the administration of HPX upon stored RBC transfusions in mice was shown to be partially effective, as discussed earlier. In the context of ICH and SAH, HPX replenishment alone exhibited deleterious effects in several preclinical studies, promoting the administration of both Hp and HPX. Furthermore, the potential benefits of Hb/HPX replacement have been highlighted in β‐thalassemia major and intermedia and hereditary spherocytosis (106).

The limited application of HPX in clinical settings can be partially attributed to its dual effects in different pathologies. For example, renal injury and resultant impairment of renal function are common complications of various hemolytic diseases. However, the effects of HPX on renal function remain to be elucidated. A study found that HPX could prevent complement activation in the kidneys of patients (or mice) with phenylhydrazine-induced hemolysis, substantiating the beneficial effects of HPX as a therapeutic agent, given that C3 deposition is associated with kidney injury (26). However, HPX does not always serve a protective role in hemolysis-associated renal injury. While HPX deficiency promotes AKI in sickle cell mice under hemolytic stress, HPX injection did not alter hemoglobinuria (24, 25). In a Shiga-toxin–induced HUS mouse model, HPX knockout mice displayed improved survival and reduced tubular iron deposition compared to wild-type mice (67).

Aside from HPX-mediated potential harm to the kidney, HPX increases the concentration of Hb in the CNS and promotes the aggregation of Hb or globin, consistent with findings observed in the kidneys. HPX-Hb increases iron-dependent neurotoxicity, which can be attenuated by Hp. These effects have also been shown to be deleterious to the brain, resulting in memory deficits in young mice (107). Therefore, HPX supplementation alone is not indicated for certain hemolytic conditions, while HPX plus Hp supplementation is more advisable when encountering toxicity mediated by iron or heme in pathological conditions. Plasmapheresis is a symptomatic treatment for autoimmune diseases and was investigated as a treatment for SCD patients with multiple organ dysfunction. In a case report, SCD patients refractory to RBC exchange were treated with plasmapheresis. Patients with a significant rise in plasma HPX and Hp levels showed clinical relief, in contrast to patients with minimal increases in HPX and Hp levels (108).

Recently, a hemopexin-Hp fusion protein with binding affinity and detoxification capacity for both heme and Hb was produced, which may contribute to the development of hemopexin-Hp therapies (109). This bifunctional fusion protein was generated using transient mammalian gene expression of Hp integrated with the pro-haptoglobin processing protease C1r-LP co-transfection for recombinant Hp-variant generation. This technology can likely be harnessed to generate multifunctional fusion proteins involved in the entire process of Hb and heme detoxification and clearance (110). Another study focused on increasing HPX yield and enhancing the interaction between heme and HPX (110). Utilizing a recombinant production strategy in human cell lines, Elena Karnaukhova et al. produced recombinant human HPX (rhHPX) by constructing an rhHPX expression plasmid and transfecting it into an HEK293 mammalian cell line (110).

The specific patient populations for which HPX-Hp replenishment is indicated and the degree of effectiveness achievable are beyond our current knowledge. Indeed, more research should be directed towards investigations into the role of HPX in various heme-related diseases and prospective clinical studies.

### Hemopexin gene therapy

Historically, early efforts in HPX replacement therapy primarily focused on protein and plasma HPX levels. However, researchers have shifted their focus in recent years toward upregulating HPX expression and generating recombinant human HPX in eukaryotic cell expression systems (111). In mouse models of SCD, upregulating the endogenous hepatic synthesis of hemopexin through gene therapy has ameliorated inflammation and vaso-occlusion. This was demonstrated by quantifying hepatic nuclear Nrf2 expression, HO-1 activity, and NF-κB levels (112). Inspired by the success of gene therapy with adeno-associated virus (AAV) in hemophilia treatment, long-term expression of human HPX (hHPX) with AAV vectors was developed (113, 114). These vectors integrated with the full cDNA sequence of hHPX to prompt continuous hHPX expression for 58 days. Subjects exposed to heme challenges induced by heme infusions and phenylhydrazine survived (114). With the application of precision medicine and genome sequencing, the relationship between HPX genes and individualized drug side effects as well as tumorigenesis needs to be further investigated (115, 116).

## Conclusion and perspectives

Heme overload is typically observed following hemolysis. Under physiological conditions, heme is quickly scavenged by the immune system in the plasma, preventing adverse pathophysiology driven by heme. HPX, one of the heme-binding proteins with the highest affinity, has broadened the therapeutic possibilities in hemolytic and thrombotic-related conditions because its administration can reverse the toxic effects of heme. This review summarized the double-edged functions of HPX in various hematological-related pathologies that cause heme overload based on current studies, such as SCD, transfusion-induced hemolysis, sepsis, ICH, SAH, atherosclerosis, and more. With an update of current preclinical and clinical studies, the review has outlined the potential of HPX as a biomarker for assessing the severity of certain diseases and its therapeutic value based on the pros and cons of its replenishment.

Initially, there has been much uncertainty due to the conflicting outcomes stemming from HPX supplementation. However, a possible hypothesis was proposed after a comprehensive understanding of HPX functions based on current studies. HPX replenishment can be protective when the heme clearance system is significantly undermined. In pathological conditions that mainly cause inflammation, HPX probably promotes precipitation and destabilization of Hb and HPX-Hb. Consequently, disassociated Hb can induce iron toxicity and oxidative stress, causing additional organ injury. Under such circumstances, HPX administration is detrimental. However, the border line between the two edges of HPX remains a scientific gap that requires further investigation. More research into the effects of HPX on different organs with heme overload due to systemic infection by fungi is expected. Additionally, ongoing investigations are being conducted to explore the diseases for which HPX administration is relevant.

In addition to its role as a therapeutic agent, new technologies for producing HPX protein using gene therapy were briefly introduced. The advancement in generating recombinant human HPX and fusion proteins offers new insights into the analysis of HPX-heme interactions and the neutralization of heme and its precursor, Hb. In this regard, the administration of a hemopexin-haptoglobin fusion protein may help avoid the deleterious effects caused by the sole administration of HPX, though further verification is needed.

Overall, the optimization of HPX therapy represents a novel and promising direction in the field of hematology. Recent progress includes upregulating endogenous HPX gene expression, producing recombinant HPX, and developing multifunctional fusion proteins with binding affinity and detoxification capacity for both heme and Hb.

## Author contributions

YL: Writing – original draft, Conceptualization, Data curation, Formal Analysis. RC: Data curation, Writing – original draft. CW: Data curation, Writing – review & editing. JD: Writing – review & editing. SL: Conceptualization, Funding acquisition, Supervision, Writing – review & editing.
